# Hospital Meetings, &c.

**Published:** 1899-04-01

**Authors:** 


### HOSPITAL MEETINGS, &C.

### WESTERN OPHTHALMIC HOSPITAL.

Sir Reginald Hanson presided at the annual general meeting of the Western Ophthalmic Hospital on Wednesday, 23rd inst. The report presented again showed an increase in the work done during the year. 132 in-patients were treated, as compared with 126 in 1897, while the number of out-patients was 26,606, as against 25,332. Notwithstanding the increase in the number of patients, the cost of establishment and administration had been reduced. In 1897 these charges amounted to over £600, including £100 cost of special repairs. Last year the amount under these heads was only £270. The ordinary income for the year was £560; the ordinary expenditure £527. The report was adopted. After some discussion the following were elected to the committee of management, on the motion of Mr. Diggle, in addition to the ex-officio members: Messrs. Browne, Diggle. Boger, Campbell, Chambers, Fayrer, Pattison, and Summers, Mr. Holthouse called attention to the need for a new building, both on account of their limited space and of the unsanitary condition of some parts. Repairs were simply so much money thrown away. He thought the time had come when an effort should be made to take the matter in hand, and proposed that a special committee should be appointed to examine and report on the resources of the hospital, and make suggestions for the carrying out of the work of erecting a new building, to inquire as to a suitable site, and as to the steps to be taken for the raising of funds. After some discussion this was carried, and a committee appointed consisting of Sir Reginald Hanson, Mr. J. R. Diggle, Mr. Browne, Mr. Holthouse, Mr. Wray, and Mr. W. H. Maw, with power to add to their number. Votes of thanks to the honorary staff and to the chairman concluded the meeting.

### LONDON HOMŒOPATHIC HOSPITAL.

The forty-ninth annual general meeting of the London Homœopathic Hospital was held on Friday, March 24th, Mr. J. P. Stilwell, the chairman of the board of management, presiding, in the absence of Lord Cawdor, the treasurer, who had been announced to take the chair, but who was unfortunately suffering from influenza.

The report for the past year was read by Mr. G. A. Cross, the secretary-superintendent. It stated that the numbers both of in-patients and out-patients had exceeded those of any previous year. The former numbered 1,111, and the latter 18,551, including 993 renewals. An investigation into the means of persons applying for treatment as out-patients was held during the year, with the result that of 313 cases inquired into five only were deemed ineligible and 17 doubtful; this practically corroborating the results of the method adopted for some years past of inquiry when apparently called for, and reference to the secretary-superintendent when in doubt. The ordinary income for the year was £6,620, a decrease of £433 as compared with 1897. The ordinary expenditure was £9,862, including £1,102 for maintenance and wages of the private nursing institute. The total deficit on current account at the end of 1898 was no less than £9,556, and this was met under the sanction of a special general meeting, by moneys which should otherwise have been invested and by the renewal of loans from the bankers of £3,000. At the close of the year the Board were gladdened by the announcement of a legacy of upwards of £11,000, residue of the estate of Dr. J. Say Clarke, of Ryde. Success was reported in the treatment of diphtheria in the newly-opened special ward, and also in regard to a new departure—the appointment as lady house physician for the gynæcological department of Miss Edith Nield, L.R.C.P.Edin.—an appointment made practicable by the special help of Sir Henry Tyler, the chairman of the House Committee. To celebrate the fiftieth year of the hospital a Jubilee dinner has been arranged for at the Hotel Cecil in June, under the presidency of the Earl of Cawdor, and a special appeal is made for funds in connection with this.

The adoption of the report was moved by the Chairman, who went over some of its salient points, and carried *nem. con.*

In connection with the bequest of Dr. Say Clarke, Sir Henry Tyler brought forward a motion to the effect that, it having come to the knowledge of the Governors that a nephew of the testator, Mr. George Aitchison, is living in great distess in Richmond, Pennsylvania, penniless, paralysed, and with a wife and child dependent on him, the committee be empowered to inquire into the matter, and to make an award if they should see fit of an annuity not exceeding £50 per annum.

This was seconded by Mr. Smart, who said that though, of course, there was no legal claim upon them, yet there was no doubt a very strong moral claim under all the circumstances.

Colonel Clifton Brown expressed a doubt as to whether they were justified in taking such action, but on being put to the meeting the motion was carried unanimously.

The retiring members of the board of management—the Earl of Cawdor, Colonel Clifton Brown, Mr. Edward Brown, Mr. James Epps, Mr. H. W. Prescott, Mr. F. G. Smart, and Mr. W. H. Trapmann—having been re-elected, and the election of Dr. Robertson Day and Dr. Washington Epps confirmed, the meeting separated with votes of thanks to the staff and to the chairman.

### UNIVERSITY COLLEGE HOSPITAL.

Lord Monkswell (the treasurer) presided at the annual general meeting of the North London or University College Hospital held on Friday, March 24th, in the board room of the well-known institution, now being rebuilt through the generosity of Sir J. Blundell Maple, M.P. The committee having been elected, the Chairman summarised the voluminous report presented, which opened with a record of the Queen's donation of £100. The site of the new hospital building is a quadrilateral area of 232 ft. by 225 ft.; the plan of the work being a diagonal cross. The ward wings each contain four large wards in as many storeys, and each 86 ft. long, 25 ft. wide, and 12 ft. 3 in. high; each will contain 24 beds, and the total beds accommodated will be nearly 300. The Chairman explained the details of a special feature, which will be the complete isolation of each ward; and stated that it was hoped some portions of the new building would be ready for use in the course of the current year. The present arrangement with the All Saints' Sisterhood for the nursing will cease this summer, and in future the nursing will be carried on under the direct control of the Hospital Committee, who are taking steps to engage a matron and a staff of nurses. The arrangement with the sisterhood has been in force since 1862, and the committee recorded their sense of the great value of the devoted services of the sisterhood to the hospital during so long a period. Mr. Walter Bailey has been appointed vice-chairman of the Hospital Committee, and Mr. Richard Horton Smith, Q.C., succeeds to the vacancy on the board caused by the resignation of Sir John Hutton. Mr. G. Nicol has been appointed as inquiry officer to interview out-patients, with a view to prevent abuse of the charity, the committee in this acting on representations from the Hospital Sunday Fund and the Central Hospital Council. The council of the Sunday Fund awarded to the hospital £1,012 from the 1898 collection, and the Saturday Fund had awarded £309 from its 1897 collection. The annual subscription of £1,400 awarded the institution by the Prince of Wales's Fund to reopen 25 of the 50 beds which were closed in 1897 for lack of funds, had been again received. The committee record a debt of £8,029 at the end of 1898; the year 1897 closed with a debt of £6,659. The expenditure for 1898 was over £18,000, whilst the income which can be relied upon does not exceed £8,000. The Chairman, in conclusion, pointed out that the sum required for furniture and additional medical and surgical appliances for the new building must be large, and it was estimated that the annual income required to fully work the new hospital when completed could not be far short of £30,000. The public seemed to be under the impression that, having received such a handsome sum for the building, that the committee were "in clover." That was not so, and although the sum he had stated might appear enormous compared with the number relieved—now over 50,000 cases in a year, to be probably increased to 60,000—it was moderate. The accounts for 1898 showed that the total ordinary income amounted to £14,427, and the total ordinary expenditure to have been £16,706; the extraordinary income was £8,610. The chairman's motion for the adoption of the report having been seconded by Mr. Henry Lucas, it was adopted; and a cordial vote of thanks to the chairman, on the motion of Mr. Horton Smith, concluded the proceedings.

### WEST-END HOSPITAL FOR DISEASES OF THE NERVOUS SYSTEM.

The annual meeting of the Governors and subscribers of the West-End Hospital for Diseases of the Nervous System, Paralysis, and Epilepsy, was held at 73, Welbeck Street, W., on Thursday, March 23rd, Mr. C. E. Porter, chairman of the committee, presiding.

The report for the year 1898, which was presented to the Governors by the committee, stated that the ordinary receipts had been £2,722, and the ordinary expenditure £2,770. The accounts showed an extraordinary expenditure of £751 on behalf of the building fund; there being a balance at the bank and in hand of £511. The liabilities amounted to £1,176, against £971 on December 31st, 1897. The committee reported that no legacies or extraordinary receipts had fallen to the hospital during the year. Further extensive improvements in the internal arrangements had been carried out in addition to the installation of the electric light already reported upon; £6,430 was now due by the building to the general fund, whereas a year ago the figures were £5,678. The grants of £82 10s. and £75 19s. from the Hospital Sunday and Saturday Funds respectively were thankfully acknowledged, the committee, however, expressing disappointment in not receiving a grant from the Prince of Wales's Fund last year (as in 1897 an amount was received, without application), but it was explained that an informality in the method of application was the cause of the hospital being overlooked in 1898. The annual subscription list, the sole reliable source of income, had steadily advanced. The committee stated that the Duchess of Devonshire was so pleased with the arrangements when visiting the hospital that her Grace promised to report to the Princess of Wales to that effect. The committee have been obliged to engage rooms outside for some of the nurses and staff, the hospital not providing sufficient accommodation.

Mr. B. Heckstall-Smith, the secretary, having dealt with the formal business of the meeting,

Dr. T. Outterson Wood, the senior physician, read the medical report. This showed that 196 patients had been admitted during the year, and that at December 31st, 1897, 44 remained, giving a total of 240. Of these, 20 were cured, 188 improved, 30 were incurable, 2 died, and none remained at December 31st last, the wards being temporarily closed owing to an outbreak of measles. The attendances for electrical treatment numbered 7,016 against 4,912, and, adding the attendances for ordinary medical treatment, the out-patients totalled no less than 29,481.

The Chairman, in moving the adoption of the report, said the annual subscriptions showed an increase of £141, being £1,195 in 1898 as against £1,053 in 1897. Donations had increased by £194, whilst patients' payments had slightly fallen off, although he hoped for an increased income from the additional patients. He called attention to the extraordinary expenditure and stated that the cost of the repairs had been met by a "nest egg" of £1,000 on deposit. He further alluded with satisfaction to the election of Mr. James Cantlie, M.B., F.R.C.S., to the medical staff in place of Mr. Edward Cotterill, who had died. The Chairman paid a warm tribute to Captain Dowell, the treasurer, who, he pointed out, had in five years almost doubled the income of the hospital.

Captain Dowell having seconded the motion, the report was unanimously adopted.

